# Metabolomic Profiling and Antioxidant Properties of Chilean *Eucryphia cordifolia* Cav.: Insights from Leaves, Flowers, and Monofloral Honey

**DOI:** 10.3390/antiox14030292

**Published:** 2025-02-28

**Authors:** Rafael Viteri, Ady Giordano, Gloria Montenegro, Mario J. Simirgiotis, Flavia C. Zacconi

**Affiliations:** 1 Departamento de Química Orgánica, Facultad de Química y de Farmacia, Pontificia Universidad Católica de Chile, Santiago 7820436, Chile; rviterie@uees.edu.ec; 2 Escuela de Ciencias Ambientales, Universidad Espíritu Santo, Guayaquil 092301, Ecuador; 3 Departamento de Química Inorgánica, Facultad de Química y de Farmacia, Pontificia Universidad Católica de Chile, Santiago 7820436, Chile; 4 Departamento de Ciencias Vegetales, Facultad de Agronomía y Sistemas Naturales, Pontificia Universidad Católica de Chile, Santiago 7820436, Chile; gmonten@uc.cl; 5 Instituto de Farmacia, Facultad de Ciencias, Universidad Austral de Chile, Campus Isla Teja, Valdivia 5090000, Chile; mario.simirgiotis@uach.cl; 6 Institute for Biological and Medical Engineering, Schools of Engineering, Medicine and Biological Sciences, Pontificia Universidad Católica de Chile, Santiago 7820436, Chile; 7 Center for Nanomedicine, Diagnostic & Drug Development (ND3), Universidad de Talca, Talca 3460000, Chile; 8 Centro de Nanotecnología y Materiales Avanzados, CIEN-UC, Pontificia Universidad Católica de Chile, Santiago 7820436, Chile

**Keywords:** monofloral honey, ulmo, HPLC-MS, quantification, chromatography, antioxidants, phenolics

## Abstract

This study aimed to characterize the metabolomic profile of monofloral honey from *Eucryphia cordifolia* (ulmo) and evaluate the potential transfer of bioactive compounds from the plant parts, including the leaves and flowers, to the honey. Using UHPLC/Q-TOF-MS analysis, various flavonoids and phenolic acids were identified and quantified in extracts from the leaves, flowers, and honey from *E. cordifolia*. Given their rich polyphenolic composition, *E. cordifolia* leaves were included in this study to assess their potential contribution to the antioxidant properties and chemical markers of ulmo honey. Additionally, the polyphenolic compounds in honey samples were quantified. Chromatographic analysis via UHPLC-MS/MS revealed that ulmo honey contains phenolic acids such as gallic, syringic, ferulic, chlorogenic, caffeic, and coumaric acid, as well as flavonoids including pinocembrin, quercetin, luteolin, kaempferol, epicatechin, apigenin, and isorhamnetin. The results indicate that pinocembrin and gallic acid are the main chemical markers of ulmo honey, while isorhamnetin could complement its characterization as a complementary marker. UHPLC/Q-TOF-MS analysis was also utilized to compare the compounds present in the honey with those found in the plant parts (leaves and flowers), respectively. A total of 10 shared compounds were identified, 9 of which were preliminarily identified, while 1 remains unknown. Notably, dihydroquercetin 3-*O*-rhamnoside, quercetin 3-*O*-rhamnoside, cyanidin 3-(*p*-coumaroyl)-glucoside, and eupatorin were detected in ulmo honey for the first time. Along with gallic acid, pinocembrin, and isorhamnetin, these compounds could contribute to a characteristic fingerprint for identifying the botanical origin of the honey. Overall, these findings provide valuable insights into the chemical composition of ulmo honey and its potential application as a functional product with antioxidant properties.

## 1. Introduction

Honey is a natural food product with a sweet taste that is produced by the honeybee (*Apis mellifera* L.) and known for its high nutritional and medicinal value. In the Egyptian and Greek cultures, it was used to treat various ailments, like stomach ulcers and skin wounds [1]. Honey is a natural matrix composed of approximately 80–85% total carbohydrates (about 40% fructose, 30% glucose, and 10–15% maltose, sucrose, and other sugars), with the exact composition largely depending on the plant species from which the nectar or exudate was collected, as well as the environmental conditions and climate [2].

**Figure 1:**
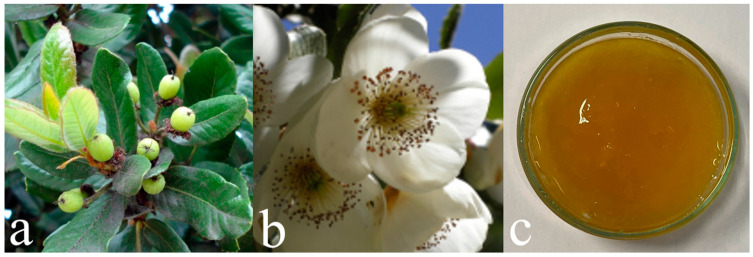
Leaves (**a**), flowers (**b**), and honey of *E. cordifolia* (**c**).

Chile ranks 29th among global honey producers with a production of 11,600 tons of honey annually (0.68% of global production), of which 10,000 tons are exported [3]. Chilean honey is highly appreciated due to its nematicidal and antifungal properties and its known chemical characteristics and biological attributes. A particular species, ulmo (*Eucryphia cordifolia* Cav.), possesses flowers that are highly appealing to bees (Figure 1). In recent times, ulmo honey has garnered recognition for its antioxidant and antimicrobial characteristics. The monofloral honey of *Eucryphia cordifolia* has been widely used in traditional medicine in the Mapuche-Chilean culture, especially for the treatment of infections. The ulmo tree is distributed in southern Chile from the Ñuble Region to the Los Lagos Region, being a typical tree of the Valdivian forest. Its flowers produce a large amount of nectar, making it an excellent honey tree and providing a product with a pleasant aroma and characteristic golden color (Figure 1) [4]. The ulmo (*E. cordifolia*) blooms between January and March, depending on the latitude and altitude. During this period, bees collect nectar from its flowers to produce honey. The ulmo honey harvest typically takes place between March and April.

Recently, a study conducted by our group reported that the leaves and monofloral honey of *E. cordifolia* exhibit antioxidant and antibacterial activities [5,6]. Another study suggests that ulmo honey may possess healing properties, along with potential anticancer, antioxidant, and antibacterial effects [4]. The presence of phenolic compounds, flavonoids, tannins, triterpenes, diterpenes, steroids, and volatile oils in the leaves and stems of *E. cordifolia* has been reported, evidencing its bioactive potential [7,8,9,10,11]. Therefore, their inclusion in this study allows for a more comprehensive evaluation of the plant’s potential impact on the composition and bioactivity of the resulting honey. However, information on the metabolomic identification of its flowers and the honey derived from this specimen is still limited. Considering that ulmo honey originates from floral nectar, it is essential to evaluate its metabolomic profile and determine whether its bioactive compounds present antioxidant properties. To date, the antioxidant properties of *E. cordifolia* flowers have not been studied, which represents a gap in knowledge about the chemistry of this species and its potential influence on honey composition.

Various techniques have been developed to verify the floral origin of honey, aiming to ensure its authenticity and distinguish it from other types of honey. Among these methodologies, melissopalynology has been widely used to identify the characteristic pollen profile of the species in question. Additionally, infrared spectroscopy and chemometric analysis have been used to predict the phenolic and flavonoid content in ulmo honey [12]. Furthermore, sensory analysis and SPME-GC-MS have enabled the identification of distinctive compounds in monofloral ulmo honey [13]. In recent years, metabolomic profiling using mass spectrometry has emerged as an advanced tool for detailed chemical characterization.

In this study, metabolomic analysis using high-resolution mass spectrometry (LC-MS/MS) with a QTOF analyzer was employed to identify and characterize flavonoids, phenolic acids, and anthocyanins in *E. cordifolia*. This technology provides a comprehensive profile of the metabolites present in ulmo honey and allows comparison with leaf and flower extracts of the species. Therefore, the main objective of this research was to evaluate the relationship between the chemical compounds present in the plant and their possible transfer to honey, aiming to identify potential chemical markers that contribute to its botanical authentication.

## 2. Materials and Methods

### 2.1. Chemicals and Reagents

Folin–Ciocalteu’s phenol reagent, Ethyl acetate, *n*-hexane, and methanol were purchased from Merck (Darmstadt, Germany). 1,1-diphenyl-2-picrylhydrazyl (DPPH), potassium persulfate (K_2_S_2_O_8_), 2,2′-azino-bis(3-ethylbenzothiazoline-6-sulphonate) (ABTS), 1,3,5-triphenyltetrazolium chloride (TPTZ), Trolox (6-hydroxy2,5,7,8-tetramethylchroman-2-carboxylic acid), FeSO_4_·7H_2_O, gallic acid, cinnamic acid, syringic acid, ferulic acid, chlorogenic acid, caffeic acid, *p*-coumaric acid, benzoic acid, pinocembrin, rutin, quercetin, luteolin, kaempferol, epicatechin, catechin, apigenin, myricetin, isorhamnetin, taxifolin, chrysin, galanganin, genistein, and hesperetin were all purchased from Sigma Chemical Co. (St. Louis, MO, USA).

### 2.2. Plant Material and Extraction

The plant material (leaves and flowers) of *E. cordifolia* was collected in January 2020 in Puerto Varas (Chile). Ethyl acetate and aqueous fractions from the methanolic extract of *E. cordifolia* leaves were previously obtained [10]. The flowers were dried at room temperature in the shade. The dry and powdered material was subsequently extracted separately with *n*-hexane, ethyl acetate, and methanol by maceration followed by filtration. The extracts were prepared at room temperature by stirring for 48 h. The extracts were concentrated to dryness in a rotary evaporator (Rotavapor^®^ R-100, BÜCHI Labortechnik AG, Flawil, Switzerland) under reduced pressure at 45 °C. Subsequently, they were stored at 4 °C for further analysis. Also, *n*-hexane was used to defat the flower samples, and the resulting extract was not included in the analysis.

### 2.3. Honey Collection

Honey samples from ulmo honey (UH055, UH056, and UH057) were obtained from local beekeepers in the Mediterranean climate zone of southern Chile (Los Lagos: Región X, Los Ríos: Región XIV). For comparison, three samples of monofloral honey (Tiaca, Guindo Santo, Alfalfa Chilota; 3 samples of each honey) were collected from southern Chile. Only honeys containing ≥ 45% ulmo pollen, as determined by melissopalynological analysis, were selected. The samples were stored at −20 °C until the study was conducted. The water content, pH value, diastase activity and sugar content of the ulmo honey samples were determined according to the methods proposed by the Association of Official Analytical Chemists (AOAC) [14].

### 2.4. Honey Extract Preparation

Ulmo honey samples (1 g) were dissolved in 10 mL of distilled water and allowed to stand for approximately 1 h. The sample was added to an Oasis HLB cartridge, which was previously conditioned with 5 mL of methanol and 5 mL of distilled water, respectively. The washing step was finished by adding 5 mL of distilled water, extracting mainly interfering sugars, and allowing the sample to dry for approximately 15 min under vacuum. The phenolic compounds were eluted with 5 mL of methanol. The methanol extract was filtered through a 0.45 μm filter and stored at −20 °C for further analysis, as described in Section 2.5 and Section 2.6 [15].

### 2.5. Quantitative UHPLC-MS/MS Chromatographic Analysis

All samples were analyzed with an ABSciex triple Quad 4500 mass spectrometer equipped with an electrospray (TurboV) interface coupled to an Eksigent Ekspert Ultra LC100 automatic sampling system with an Ekspert Ultra LC100-XL (AB/Sciex Concord, Concord, ON, Canada). A LiChrospher 100 RP-18 (125 mm × 4 mm id, 5 µm) column was utilized, and 0.1% formic acid in water (A) and methanol (B) with the following gradient compositions were used as a mobile phase: 0–1 min, 15% B; 1–17 min, 15–100% B; 17–21 min 100–100% B; 21–22 min, 100–15% B; 22–25 min, 15–15% B. Ten microliters of each sample were injected at a flow rate of 0.5 mL/min at 30 °C. Calibration curves were recorded for each compound using available standards in the 10–250 μg/L range.

### 2.6. UHPLC/Q-TOF-MS/MS

Chromatographic profiling was performed on an Agilent 1260 Infinity UHPLC system (Agilent, Waldbronn, Germany) coupled to a Triple TOF 5600 QTOF-MS detector (AB SCIEX, Toronto, ON, Canada) with an electrospray ionization (ESI) source. Data acquisition and processing were performed using the Analyst TF 1.5, PeakView™ 1.2, and LibraryView™ (AB Sciex, Toronto, ON, Canada) software, respectively. For analysis, 2 mg per mL of the plant parts was dissolved in distilled water/methanol (1:1 *v*/*v*) and then 5 µL of the filtered solution (PTFE filter, Merck KGaA, Darmstadt, Germany) was injected.

Liquid chromatography was performed using an Acquity UPLC C18 1.7 μm, 2.1 mm × 50 mm column (Waters Corporation, Milford, MA, USA). A gradient of 0.1% formic acid (solvent A) and methanol with 0.1% formic acid (solvent B) was used as follows: between 0 and 13 min, 90% (A) and 10% (B), then between 13 and 15 min, 100% (B), and, finally, between 15 and 22 min, 90% (A) and 10% (B). The flow rate was adjusted to 0.4 mL/min and the separation was finished in 35 min.

MS data were recorded from 80 to 1200 *m*/*z* in negative mode and directed information dependent acquisition (IDA) analysis was performed in survey scan type (TOF-MS) using dependent scan (production) with −50 V collision energy voltage. The temperature was 400 °C with 25 psi curtain gas (CU), while 50 psi was applied for both ion source gas 1 (GC1) and ion source 2 gas (GS2). The ion spray voltage was −4500 V, the disaggregation potential was (DP) 90 V, and the collision energy voltage was set to (CE) −50 V (Supplementary Materials, Figures S1–S5).

### 2.7. Antioxidant Activity Assays

#### 2.7.1. 2,2-Diphenyl-1-picrylhydrazyl Radical (DPPH)

The DPPH• radical was determined by the decolorization method [16] and adapted to 96-well plates. Briefly, 50 µL of sample was mixed with 150 µL of a DPPH solution (0.15 mM) in methanol and kept in the dark for 30 min. Subsequently, the absorbance was measured at 517 nm in a microplate reader (Cytation™ 5, BioTek, Santa Clara, CA, USA). The results are expressed in TEAC, the antioxidant activity equivalent to Trolox (μmol Trolox/g extract). The synthetic antioxidant reference, Trolox, at a concentration of 25–200 µM in methanol solution, was tested under the same conditions.

#### 2.7.2. 2,2′-Azino-bis(3-ethylbenzothiazoline-6-sulphonate) (ABTS)

The ABTS assay was carried out by means of the iron reduction method [17], and adapted to 96-well plates. A stock solution of ABTS was prepared by mixing ABTS and potassium persulfate (K_2_S_2_O_8_) in distilled water, considering a final concentration of 7 mM for ABTS and a final concentration of 3.6 mM for K_2_S_2_O_8_. The reaction was then incubated at room temperature for 24 h in the dark. Subsequently, the ABTS stock solution was diluted to a final concentration of 156 µM to obtain a final absorbance of 0.70 ± 0.02 at 732 nm. Then, 50 µL of sample solution was mixed with 150 µL of reagent solution and the absorbance was measured at 732 nm after 30 min in the dark using a microplate reader (Cytation™ 5, BioTek, Santa Clara, CA, USA). The calibration curve was built with Trolox, and the results were expressed as micromoles of Trolox equivalents per gram of dry extract (µmol TE/g).

#### 2.7.3. Ferric Ion Reducing Antioxidant Power Assay (FRAP)

The FRAP assay was performed according to the reported procedure [18]. Briefly, the FRAP reagent was prepared in situ by mixing 300 mM acetate buffer (pH 3.6), a solution of 10 mM 2,4,6-tripyridyl-s-triazine (TPTZ) in 40 mM hydrochloric acid and 20 mM ferric chloride in a 10:1:1 ratio (*v*/*v*/*v*). Then, 20 µL of each extract was mixed with 180 µL of FRAP reagent in 96-well plates. The mixture was left for 30 min in the dark and the absorbance at 595 nm was measured in a microplate reader (Cytation™ 5, BioTek, Santa Clara, CA, USA). Ferrous sulfate heptahydrate (FeSO_4_·7H_2_O) was used as standard to perform the calibration curve and results were expressed as millimoles of Fe per gram of dry sample (mmol Fe/g).

### 2.8. Total Phenolic Content (Folin-Ciocalteau)

The phenolic content was analyzed as previously reported [18]. A total of 20 µL of each sample was mixed with 100 µL of Folin–Ciocalteau reagent (1:10 *v*/*v*) and 80 µL of a Na_2_CO_3_ solution (7.5%), then incubated for 60 min at room temperature, after which the absorbance of the resulting blue solution was measured at 760 nm in a microplate reader (Cytation™ 5, BioTek, Santa Clara, CA, USA). The obtained absorbance values were replaced in the equation of the standard curve of gallic acid. The results of the total phenol content are expressed in mg of gallic acid equivalent per gram of dry extract (mg GAE/g).

### 2.9. Statistical Analysis

All assays were conducted in triplicate, with the results being expressed as the median ± standard deviation (SD) using Microsoft Excel (Microsoft 365, Microsoft Corporation, Redmond, WA, USA). Statistical differences between groups were considered significant at *p* < 0.05 and were analyzed using one-way ANOVA followed by Tukey’s post hoc test in the commercial software Minitab 19.

## 3. Results

### 3.1. Identification of Compounds in E. cordifolia Extracts by LC QTOF-MS/MS

In this study, the fingerprint was generated by LC-QTOF-MS/MS, which allowed for the determination of several families of compounds in the extracts of the leaves, flowers, and honey of *E. cordifolia*. The metabolomic study of the three ulmo honey samples enabled the identification and characterization of their chemical profiles, highlighting the compounds present in each. For the construction of the compound table, only metabolites shared among the three samples were considered, ensuring a more consistent and representative depiction of the characteristic chemical profile of *E. cordifolia honey*. The tentative identification of the metabolites was carried out by the analysis of full scan mass spectra, base peak chromatograms, and fragmentation patterns in comparison to the PubChem database, FooDB, and data from the literature.

#### 3.1.1. Identification of Metabolites in the Ethyl Acetate Fraction of Leaves

Eighteen compounds were detected, and 14 compounds were tentatively identified in the ethyl acetate fraction (EtOAcF) from the methanolic extract of *E. cordifolia* leaves. Of these compounds, two are phenolic acids (1, 15), eight are flavonoids (5, 6, 7, 8, 9, 10, 11, and 18), three are anthocyanins (12, 13 and 14), and one is a polyphenolic compound (17); four compounds remained unidentified, respectively (2, 3, 4, and 16) (Table 1, Supplementary Materials, Figure S1).

##### Anthocyanin

Peak 12, with a pseudomolecular ion at M+H^+^ at *m*/*z*: 611.1300, was identified as delphinidin 3-*O*-(6-*p*-coumaroyl-glucoside) (C_30_H_27_O_14_^+^). Peak 13 exhibited a molecular ion at M+H^+^ with *m*/*z*: 578.1355 and showed a major fragment with *m*/*z*: 271.8191 that may correspond to pelargonidin; this compound could be tentatively identified as Pelargonidin-3-*O*-beta-*D*-*p*-coumaroylglucoside (C_30_H_26_O_12_^+^). Peak 14 showed a molecular ion at M+H^+^ with *m*/*z* 595.1306 and produced a major fragment with *m*/*z* 286.8026 (cyanidin); this compound was identified as cyanidin 3-(*p*-coumaroyl)-glucoside (C_30_H_26_O_13_^+^).

##### Flavonoids

Peak 5, with a M−H^−^ ion at *m*/*z*: 449.1087, was identified as dihydroquercetin 3-*O*-rhamnoside (C_21_H_21_O_11_^−^) and peaks 6–11 were identified as quercetin 3-*O*-glucoside (C_21_H_19_O_12_^−^), hesperidin (C_28_H_33_O_15_^−^), quercetin 3-*O*-pentoside (C_20_H_17_O_11_^−^), quercetin 3-*O*-rhamnoside (C_21_H_19_O_11_^−^), isorhamnetin (C_16_H_11_O_7_^−^), and kaempferol 3-*O*-rhamnoside (C_21_H_19_O_10_^−^), respectively. Finally, peak 18 was identified as eupatorin (C_18_H_15_O_7_^−^).

##### Phenolic Acids

Peak 1 was identified as galloyl glucose (C_13_H_15_O_10_^−^), and peak 15 as vanillic acid (C_8_H_7_O_4_^−^).

##### Other Polyphenols

Peak 17, with a pseudomolecular ion at *m*/*z*: 149.0971, was identified as carvacrol (C_10_H_13_O^−^).

##### Unknown

Peaks 2, 3, 4, and 16 remain unidentified to date.

#### 3.1.2. Identification of Metabolites in the Aqueous Fraction of Leaves

Twelve compounds were detected in this fraction and 10 compounds were tentatively identified in the aqueous fraction (AqF) from the methanolic extract of *E. cordifolia* leaves. Of these, two are procyanidins (1, 2), five are flavonoids (3, 4, 5, 6, 7), one is a phenolic acid (8), one is an anthocyanin (9), one is a polyphenol (12), and two compounds remain unidentified (10 and 11) (Table 2, Supplementary Materials, Figure S2).

##### Anthocyanin

Peak 9 showed a molecular ion at M+H^+^ with *m*/*z* 595.1297 and was identified as cyanidin 3-(*p*-coumaroyl)-glucoside (C_30_H_26_O_13_^+^).

##### Flavonoids

Peak 3, with a M−H^−^ ion at *m*/*z*: 449.1085, was identified as dihydroquercetin 3-*O*-rhamnoside (C_21_H_21_O_11_^−^), and peaks 4–7 were identified as quercetin 3-*O*-glucoside (C_21_H_19_O_12_^−^), hesperidin (C_28_H_33_O_15_^−^), quercetin 3-*O*-pentoside (C_20_H_17_O_11_^−^), and quercetin 3-*O*-rhamnoside (C_21_H_19_O_11_^−^), respectively.

##### Phenolic Acids

Peak 8 was identified as vanillic acid (C_8_H_7_O_4_^−^).

##### Procyanidins

Peak 1, with a pseudomolecular ion at *m*/*z* 577.1366, was identified as procyanidin dimer B1 (C_30_H_25_O_12_^−^). Peak 2 showed a deprotonated molecule at *m*/*z* 865.2006 and was identified as an isomer of a procyanidin trimer EEC with a MW of 866.

##### Other Polyphenols

Peak 12, with a pseudomolecular ion at *m*/*z*: 149.0971, was identified as carvacrol (C_10_H_13_O^−^).

##### Unknown

Peaks 10 and 11 remain unidentified.

#### 3.1.3. Identification of Metabolites in the Ethyl Acetate Extract of Flowers

Twenty-seven compounds were detected, and 21 compounds were tentatively identified in the ethyl acetate extract (EtOAcE) of *E. cordifolia* flowers. Of these, 2 compounds are anthocyanins (21 and 23), 10 are flavonoids (5, 10, 13, 14, 15, 16, 17, 18, 19, 27), 5 are phenolic acids (1, 2, 7, 8 and 24), 2 are procyanidins (6, 12), and 2 are polyphenol (4 and 26). Finally, six unidentified compounds (3, 9, 11, 20, 22, and 25) were observed (Table 3, Supplementary Materials, Figure S3).

##### Anthocyanin

Peak 21 exhibited a molecular ion [M + H]^+^ with *m*/*z*: 578.1355 and produced a major fragment at *m*/*z*: 271.8189 that may correspond to pelargonidin; this compound could be tentatively identified as Pelargonidin-3-*O*-beta-D-*p*-coumaroylglucoside (C_30_H_26_O_12_^+^). Peak 23, with a pseudomolecular ion at *m*/*z*: 624.1455, was identified as Petunidin 3-*O*-(6-*p*-coumaroyl-glucoside) (C_31_H_29_O_14_^+^).

##### Flavonoids

Peak 5, with a [M−H]^−^ ion at *m*/*z*: 289.0717, was identified as (-)-catechin (C_15_H_13_O_6_^−^); peak 10, showing a pseudomolecular ion at *m*/*z*: 447.1039, was identified as the isorhamnetin 3-*O*-glucoside (C_22_H_21_O_12_^−^). Peaks 13–19 were identified as flavonol, flavononol, and flavone derivatives, respectively. Peak 13 showed a pseudomolecular ion at *m*/*z*: 449.1087 and was identified as the dihydroquercetin 3-*O*-rhamnoside (C_21_H_21_O_11_^−^). Peak 14 (*m*/*z*: 463.0881, [M−H]^−^) was consistent with the molecular formula (C_21_H_19_O_12_^−^), corresponding to quercetin 3-*O*-glucoside. Peak 15, with a parent ion at *m*/*z*: 433.0775, was identified as quercetin 3-*O*-pentoside (C_20_H_17_O_11_^−^). Peak 16 produced a molecular ion at *m*/*z* 447.0929 and MSn ions at *m*/*z* 300.7869 (M-rhamnose), and was identified as quercetin 3-*O*-rhamnoside (C_21_H_19_O_11_^−^). Peak 17, with a [M−H]^−^ ion at *m*/*z* 315.0507, was tentatively identified as isorhamnetin (C_16_H_11_O_7_^−^). Peaks 18 and 19 were identified as kaempferol derivatives. Peak 18 produced a molecular ion at *m*/*z* 417.0824 and a kaempferol MS2 fragment at *m*/*z* 283.7958, which pointed to the presence of kaempferol 3-*O*-arabinoside (C_20_H_17_O_10_^−^). Peak 19, with a pseudomolecular ion at *m*/*z* 431.0986, which in turn produced a kaempferol ion at *m*/*z* 284.8083 (M-hexose), was proposed to be an *O*-glycosylated kaempferol derivative (kaempferol 3-*O*-rhamnoside). Finally, peak 27 was identified as eupatorin (C_18_H_15_O_7_^−^).

##### Phenolic Acids

Peak 1 was identified as 3-feruloylquinic acid (C_17_H_19_O_9_^−^), peak 2 as galloyl glucose (C_13_H_15_O_10_^−^), peak 7 as ferulic acid 4-*O*-glucoside (C_16_H_19_O_9_^−^), peak 8 as 5-*p*-coumaroylquinic acid (C_16_H_17_O_8_^−^), and peak 24, with pseudomolecular ions at *m*/*z*: 167.0350, was identified as vanillic acid (C_8_H_7_O_4_^−^).

##### Procyanidins

Peak 6 showed a deprotonated molecule at *m*/*z* 865.1995 and was identified as an isomer of a procyanidin trimer EEC with a MW of 866. Peak 12, with a [M−H]^−^ ion at *m*/*z*: 591.1508, was identified as Bis-8,8-Catechinylmethane (C_31_H_27_O_12_^−^).

##### Other Polyphenols

Peak 4 was identified as resorcinol (C_6_H_5_O_2_^−^) and peak 26, with a pseudomolecular ion at *m*/*z*: 149.0971, was identified as carvacrol (C_10_H_13_O^−^).

##### Unknown

Peaks 3, 9, 11, 20, 22, and 25 remain unidentified.

#### 3.1.4. Identification of Metabolites in the Methanolic Extract of Flowers

Twenty-six compounds were detected, and 23 compounds were tentatively identified in the methanolic extract (MeOHE) of *E. cordifolia* flowers. Of these, 3 are anthocyanins’ compounds (18, 20, and 22), 13 are flavonoids (3, 9, 10, 11, 12, 13, 14, 15, 16, 21, 23, 24, 26), 4 are phenolic acids (1, 4, 6, 7), 2 are polyphenols (2, 19), and one is a procyanidin (5). Finally, three compounds remained unidentified (8, 17, and 25) (Table 4, Supplementary Materials, Figure S4).

##### Anthocyanin

Peak 18, with a pseudomolecular ion at *m*/*z*: 610.1299, was identified as delphinidin 3-*O*-(6-*p*-coumaroyl-glucoside) (C_30_H_26_O_14_^−^). Peak 20 exhibited a molecular ion at M+H^+^ with *m*/*z*: 578.1359 and produced a major fragment with *m*/*z*: 270.8202 that may correspond to pelargonidin; this compound could be tentatively identified as Pelargonidin-3-*O*-beta-*D*-*p*-coumaroylglucoside (C_30_H_26_O_12_^+^). Peak 22, showing a pseudomolecular ion at *m*/*z*: 625.1455, was identified as petunidin 3-*O*-(6-*p*-coumaroyl-glucoside) (C_31_H_29_O_14_^+^).

##### Flavonoids

Peak 3, with a [M−H]^−^ ion at *m*/*z*: 289.0717, was identified as (-)-catechin (C_15_H_13_O_6_^−^). Peaks 9 and 10 were identified as dihydroquercetin derivatives. Peak 9 produced a molecular ion at *m*/*z* 449.1087, which pointed to the presence of dihydroquercetin 3-*O*-rhamnoside (C_21_H_21_O_11_^−^). Peak 10, with a pseudomolecular ion at *m*/*z* 465.1039, which in turn produced a dihydroquercetin ion at *m*/*z* 301.7841 (M-hexose), was proposed to be an O-glycosylated dihydroquercetin derivative (Dihydroquercetin 3-*O*-glucoside). Peaks 11–13 were identified as flavonol derivatives. Peak 11, with a parent ion at *m*/*z*: 463.0881, was identified as quercetin 3-*O*-glucoside (C_21_H_19_O_12_^−^); peak 12 showed a pseudomolecular ion at *m*/*z*: 433.0775 and was tentatively identified as quercetin 3-*O*-pentoside (C_20_H_17_O_11_^−^); and peak 13 produced a molecular ion at *m*/*z* 447.0929 and MSn ions at *m*/*z* 300.7869 (M-rhamnose), and was identified as quercetin 3-*O*-rhamnoside (C_21_H_19_O_11_^−^). Peak 14, with a [M−H]^−^ ion at *m*/*z* 315.0509, was identified as isorhamnetin (C_16_H_11_O_7_^−^). Peaks 15 and 16 were identified as kaempferol derivatives. Peak 15 produced a molecular ion at *m*/*z* 477.1036 and was identified as isorhamnetin 3-*O*-glucoside (C_22_H_21_O_12_^−^). Peak 16, with a pseudomolecular ion at *m*/*z* 431.0986, which in turn produced a kaempferol ion at *m*/*z* 284.8083 (M-hexose), was proposed to be an O-glycosylated kaempferol derivative (kaempferol 3-*O*-rhamnoside, C_21_H_19_O_10_^−^). Finally, peaks 21 and 24 were identified as the flavanone aglycones naringenin and pinocembrin, while peaks 23 and 26 were identified as the flavone aglycones apigenin and eupatorin, respectively. MS-MS analysis of all of these compounds showed characteristic daughter ions (Table 4).

##### Phenolic Acids

Peak 4 was identified as ferulic acid 4-*O*-glucoside (C_16_H_19_O_9_^−^), peak 6 as 5-*p*-coumaroylquinic acid (C_16_H_17_O_8_^−^), and peak 7 as *p*-coumaric acid (C_9_H_7_O_3_-). Additionally, peak 1 was identified as gallic acid (C_7_H_5_O_5_^−^).

##### Procyanidins

Peak 5, with a [M−H]^−^ ion at *m*/*z*: 591.1508, was identified as Bis-8,8-Catechinylmethane (C_31_H_27_O_12_^−^).

##### Other Polyphenols

Peak 2 was identified as resorcinol (C_6_H_5_O_2_^−^) and peak 19 as homovanillyl alcohol (C_9_H_11_O_3_^−^).

##### Unknown

Peaks 8, 17, and 25 remain unidentified.

#### 3.1.5. Identification of Metabolites in the Honey Extract

The three samples that were evaluated contained between 75% and 77% *E. cordifolia* pollen grains. According to the Codex Alimentarius [33], the ulmo honey in this study has a high quality, with a pH of 4.5, a water content of 19.3%, a reducing sugar content of 81.3%, and a relatively high diastase activity of 23.1 units according to Schade. Twenty-four compounds were detected, and 22 compounds were tentatively identified in the honey extract. Of these, 4 are polyphenolic compounds (1, 2, 8, 9), 15 are flavonoids (3, 4, 5, 7, 10, 12, 13, 14, 15, 17, 18, 19, 20, 22, 24), 2 are coumarins (6, 16), and 1 is an anthocyanin (21). Finally, two compounds remained unidentified (11 and 23) (Table 5, Supplementary Materials, Figure S5).

##### Anthocyanin

Peak 21 showed a molecular ion at M+H^+^ with *m*/*z* 595.1297 and produced a major fragment with *m*/*z* 286.8026 (cyanidin) and was identified as cyanidin 3-(*p*-coumaroyl)-glucoside (C_30_H_26_O_13_^+^).

##### Coumarin

Peak 6, with a pseudomolecular ion at *m*/*z*: 177.0558, was identified as mellein (C_10_H_9_O_3_^−^), and peak 16, with a pseudo-molecular ion at *m*/*z*: 161.0245, was identified as umbelliferone (C_9_H_5_O_3_^−^).

##### Flavonoids

Peak 3, with a M−H^−^ ion at *m*/*z*: 625.1413, was identified as myricetin 3-*O*-rutinoside (C_27_H_29_O_17_^−^), and peaks 4–5 were identified as quercetin 3-*O*-rutinoside (C_27_H_29_O_16_^−^) and dihydroquercetin 3-*O*-rhamnoside (C_21_H_21_O_11_^−^). Peak 7, with a [M−H]^−^ ion at *m*/*z* 447.0920 and MS2 ions at *m*/*z* 300.7859 (M-rhamnose), was tentatively identified as quercetin 3-*O*-rhamnoside (C_21_H_19_O_11_^−^), while peak 10 was assigned to quercetin (C_16_H_11_O_4_^−^). Peaks 12–15 were identified as formononetin (C_16_H_11_O_4_^−^), isorhamnetin (C_16_H_11_O_7_^−^), naringenin (C_15_H_11_O_5_^−^), and kaempferide (C_16_H_11_O_6_^−^), respectively. Peaks 17–18 were assigned to flavone cirsimaritin (C_17_H_13_O_6_^−^) and 3,7-dimethylquercetin (C_17_H_13_O_7_^−^), while peak 19 was assigned to flavanone pinocembrin (C_15_H_11_O_4_^−^) and peak 20 to daidzein (C_15_H_9_O_4_^−^). Finally, peak 22 was assigned to flavone apigenin (C_15_H_9_O_5_^−^) and peak 24 was identified as eupatorin (C_18_H_15_O_7_^−^), respectively.

##### Other Polyphenols

Peak 1 was identified as *p*-anisaldehyde (C_8_H_7_O_2_^−^) and peak 2 as syringaldehyde (C_9_H_9_O_4_^−^). Peaks 8–9 were assigned to eugenol (C_10_H_11_O_2_^−^) and homovanillyl alcohol (C_9_H_11_O_3_^−^), respectively.

##### Unknown

Peaks 11 and 23 remain unidentified.

### 3.2. Quantification of Phenolic Compounds

Phenolic acids and flavonoids are key contributors to the color, aroma, flavor, and health benefits of honey. As shown in Table 6, 22 phenolic standards were employed to quantify their presence in fractions and extracts obtained from the leaves, flowers, and honey of ulmo.

The results in Table 6 show significant differences (*p* < 0.05) in the concentrations of phenolic and flavonoid compounds among the *E. cordifolia* extracts and ulmo honey samples. Statistical analysis using one-way ANOVA followed by Tukey’s test revealed that the methanolic extract of flowers had the highest concentration of gallic acid (1378.69 ± 0.38 mg/kg), which was significantly higher than all other fractions/extracts and honey samples that were analyzed. In contrast, in ulmo honey, the concentration of this compound ranged from 3.72 ± 0.16 mg/kg to 32.80 ± 0.20 mg/kg, suggesting a possible transfer from the plant to the honey in lower proportions. Pinocembrin was one of the most abundant flavonoids in the ulmo honey, with sample UH056 showing the highest concentration (1196.53 ± 0.24 mg/kg), followed by sample UH057 (231.44 ± 0.09 mg/kg). These differences were statistically significant (*p* < 0.05) and suggest variability in the chemical composition of the honey, which is possibly influenced by environmental and floral factors. In the plant extracts, pinocembrin was found at a higher concentration in the methanolic extract of the flowers (123.24 ± 0.15 mg/kg), supporting its potential botanical origin in *E. cordifolia*. Other compounds, such as isorhamnetin and quercetin, also showed significant differences among the samples. Isorhamnetin was found at higher concentrations in the leaf fractions (42.55 ± 0.06 mg/kg) compared to the honey, where it ranged from 1.16 ± 0.02 to 2.89 ± 0.02 mg/kg. Meanwhile, quercetin was more abundant in the methanolic extract of the flowers (3.57 ± 0.02 mg/kg) and was detected only in low concentrations in the honey samples (≤0.06 mg/kg). Overall, the variability in the concentration of these compounds suggests that, while some metabolites are transferred from *E. cordifolia* to honey, the proportion will be significantly lower, possibly due to degradation processes or chemical modifications during honey production. Taken together, these results indicate that pinocembrin and gallic acid can be considered the main chemical markers of ulmo honey, while isorhamnetin may complement their characterization. Notably, the leaves of *E. cordifolia* were found to be a rich source of polyphenolic compounds, underscoring their potential influence on the chemical profile and antioxidant properties of ulmo honey. This highlights the importance of considering not only the floral nectar but also other plant parts when evaluating the bioactive composition of honey. However, further studies are needed to assess their stability and specificity in larger samples of *E. cordifolia* honey.

### 3.3. Antioxidant Activity and Total Phenolic Content

The fractions and extracts of leaves and flowers were evaluated in vitro to determine their antioxidant activity using the DPPH, ABTS, and FRAP methods and are expressed as µmol Trolox/g of dry extract (Table 7). In addition, the total phenolics content was determined by the Folin–Ciocalteau method. Our results showed that the EtOAc and aqueous leaf fractions were more potent antioxidants than the EtOAc and MeOH extracts of *E. cordifolia* flowers. On the other hand, the ulmo 056 honey (UH056) showed the highest TPC value (58 mg GAE/100 g honey), while the ulmo 057 (UH057) honey presented the lowest TPC value (35 mg GAE/100 g honey). The values of antioxidant capacity, evaluated by the DPPH, ABTS, and FRAP methods, were between 94 (UH055) and 187 μmol TE/g ulmo honey (UH057) for DPPH, between 75 (UH055) and 122 μmol TE/g UH (UH057) for ABTS, and between 0.23 (UH056) and 0.59 0 mmol Fe/g UH (UH057) for FRAP. The results show statistically significant differences (*p* < 0.05) among the leaf fractions, flower extracts, and honey samples in terms of their antioxidant capacity and total phenolic content. Analysis of variance (ANOVA), followed by Tukey’s test, confirmed that the flower extracts, particularly those obtained with methanol, exhibited the highest antioxidant activity in the DPPH and ABTS assays, with significantly higher values than the leaf fractions and honey samples. Similarly, the total phenolic content of the flower extracts was significantly higher compared to that of the honey (*p* < 0.05), suggesting a greater concentration of bioactive compounds in the plant. Among the honey samples that were analyzed, sample UH057 showed the highest antioxidant capacity, significantly differing from the other honey samples (UH55 and UH056). These differences suggest that the chemical composition of ulmo honey may be influenced by environmental and floral factors, highlighting the need for further studies to correlate the presence of specific metabolites with its botanical origin and biological functionality.

## 4. Discussion

Honey has been consumed by humans since ancient times, mainly due to its high nutritional and medicinal values. In addition, honey is a mixture of hundreds of compounds that are found in very low concentrations, e.g., polyphenols, which give the different varieties of honey different colors, smells, flavors, and bioactivities. According to their geographical origin and floral source, the trace compound composition of honey differs considerably [35]. In this sense, it is necessary to identify chemical markers that can serve to distinguish the individual characteristics of honey.

A metabolomic study is a qualitative and quantitative analytical approach to detecting metabolites in different biological samples. The use of targeted metabolomics for quantitative analysis provides comprehensive information on biological samples. While previous studies have reported the presence of both volatile and non-volatile/semi-volatile compounds in ulmo honey, no studies have examined the chemical composition of its floral source. This study represents the first metabolomic profiling of *E. cordifolia*, providing extensive and detailed identification of the compounds present in its flowers, leaves, and honey.

**Figure 2:**
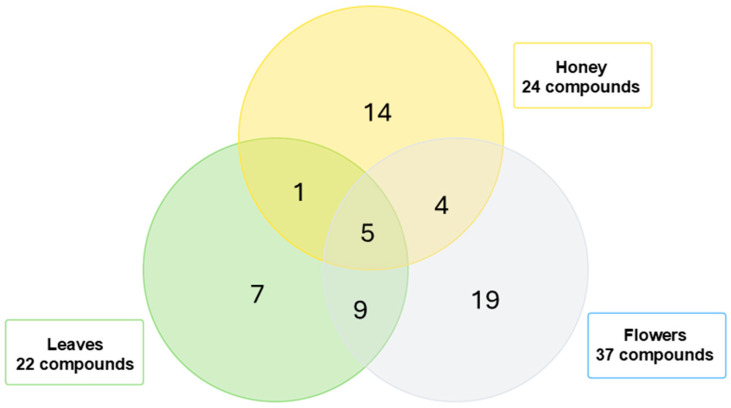
Venn diagram illustrating the shared and unique metabolites identified in the leaves, flowers, and honey of *Eucryphia cordifolia* through metabolomic analysis.

Using the established methods, the honey extract and the fractions and extracts of leaves and flowers of *E. cordifolia* were analyzed by UHPLC-Q-TOF-MS. Analysis by LC-MS resulted in 24 tentative compound identifications in the honey, 22 in the leaf fractions, and 37 in the flower extracts of *E. cordifolia*. The Venn diagram in Figure 2 compares the metabolic profiles of these samples, revealing 10 compounds shared between the plant parts and the honey, among which 5 were common to both the leaves and flowers. These shared compounds were further scrutinized to identify potential botanical origin markers. Among the six candidate compounds that were present in the leaves, five were successfully identified through exact mass and high-resolution MS2 fragmentation patterns, compared with chromatographic data and [M−H]^−^ data reported in the literature, as dihydroquercetin 3-*O*-rhamnoside, quercetin 3-*O*-rhamnoside, isorhamnetin, cyanidin 3-(*p*-coumaroyl)-glucoside, and eupatorin. On the other hand, nine were found to be common in flowers and honey, of which five were also shared with the leaves, as mentioned above. The remaining four compounds were identified as homovanillyl alcohol, naringenin, apigenin, and pinocembrin. Due to the shared presence of these compounds in both the plant parts and ulmo honey, and their absence in other honeys produced in the same region, the compounds dihydroquercetin 3-*O*-rhamnoside (astilbin), quercetin 3-*O*-rhamnoside (quercitrin), isorhamnetin, and eupatorin are potential candidates as chemical markers for ulmo honey (*E. cordifolia*).

The five flavonoids quercitrin, astilbin, isorharmnetin, cyanidin 3-(*p*-coumaroyl)-glucoside, and eupatorin are present in the ulmo honey analyzed herein and have not been reported to be simultaneously present in any type of Chilean honey to date.

On the other hand, the identification of quercitrin and astilbine in honey agrees with their reported isolation from the leaves of *E. cordifolia* [10]. As an additional contribution to the research, a set of phenolic compounds was quantified in the honey and flowers of *E. cordifolia*, along with an analysis of its leaves. This complementary analysis provided a broader perspective on the chemical composition of the species, exploring the potential transfer of metabolites from the plant to its honey. Although the primary objective of this study was to obtain a metabolomic profile of the honey of *E. cordifolia* and its relationship with the compounds present in the plant, the evaluation of phenolic compounds in the leaves offered valuable insights into the chemical variability of the species and its potential influence on the honey produced from its nectar. The identification of isorhamnetin by UPLC-MS/MS validated the findings obtained through UHPLC/Q-TOF-MS, providing additional confirmation that the presence of these compounds in honey originates from the *E. cordifolia* species.

The results in Table 6 highlight the variability in the composition of phenolic compounds of *E. cordifolia* extracts and the different honeys analyzed. Compounds such as gallic acid and pinocembrin were found at significantly higher concentrations in the ulmo honeys, particularly in sample UH056, which contained 1196.53 mg/kg of pinocembrin, suggesting the potential of this compound as a distinctive chemical marker for this variety. In contrast, compounds such as quercetin and ferulic acid were present in lower but still detectable concentrations, indicating their contribution to the unique chemical profile of each sample. This variability reflects the influence of the botanical and geographical origins of honey on its chemical composition and underscores the value of metabolomic analysis for their characterization. Previously, gallic acid, caffeic acid, coumaric acid, pinocembrin, chrysin, quercetin, abscisic acid, luteolin, and apigenin have been detected in *E. cordifolia* honey [6]. It is important to highlight that the polyphenols identified in ulmo honey have biological activity. For example, gallic acid has been reported to have antimicrobial, anticancer, and antiviral activities. Pinocembrin is a characteristic flavonoid of propolis and has been previously detected in honey from *E. cordifolia* [6] and European honey samples [36]. In addition, pinocembrin has shown anti-inflammatory, anti-asthmatic, and antitumor activities [37]. Similarly, isorhamnetin exerts beneficial effects in the areas of cardiovascular and cerebrovascular protection, antitumor activity, anti-inflammatory action, antioxidant properties, organ protection, obesity prevention, etc. Table 7 demonstrates that both *E. cordifolia* fractions and honeys analyzed herein exhibit antioxidant activity, which is closely correlated with their total phenol content [38]. The ethyl acetate and methanolic extracts of *E. cordifolia* flowers stood out for their high antioxidant activity, particularly in the DPPH and ABTS assays, with values ranging from 240.79 to 279.46 µmol TE/g DE in the DPPH and from 182.73 to 185.25 µmol TE/g DE in the ABTS assay, respectively. Meanwhile, the ulmo honeys exhibited moderate antioxidant activity, with sample UH057 showing superior performance in the DPPH and ABTS assays, which is potentially attributable to its higher content of bioactive compounds such as pinocembrin. These findings highlight the significance of phenolic compounds in determining the bioactivity of honeys and extracts, emphasizing their functional potential and their role in differentiating bee products.

## 5. Conclusions

This study contributes to the understanding of the metabolomics of the *Eucryphia cordifolia* plant, an endemic species of Chile. Chilean ulmo is an important source of monofloral honey with antioxidant properties. The three samples analyzed in this study feature various molecules from different plant parts and honey, including 22 compounds from the leaf fractions, 37 from the flower extracts, and 24 in the respective honeys of *E. cordifolia*. By examining and comparing the chemical profiles of ulmo honey and flowers using untargeted metabolomics, we report for the first time that dihydroquercetin 3-*O*-rhamnoside (astilbine), quercetin 3-*O*-rhamnoside (quercitrin), isorharmnetin, cyanidin 3-(*p*-coumaroyl)-glucoside, and eupatorin could serve as chemical markers. Notably, the leaves of *E. cordifolia* displayed a diverse and bioactive metabolomic profile, contributing significantly to the species’ overall chemical composition. Their rich contents of flavonoids, phenolic acids, and anthocyanins suggest that they may play an indirect but relevant role in the antioxidant potential of ulmo honey. These compounds could potentially act as chemical markers of floral origin for the honey of this species; however, a larger number of honey samples should be analyzed to determine a correlation between the compounds existing in the plant parts and honey of *E. cordifolia*.

## Figures and Tables

**Table 1:** Identification of metabolites in *EtOAc* fraction of *E. cordifolia* leaves.

Peak	Retention Time (min)	Tentative Identification	Molecular Formula	Theoretical Mass (*m*/*z*)	Measured Mass [M−H]^−^ or [M−H]^+^ (*m*/*z*)	Accuracy (ppm)	MS^n^ Ions	Reference
1	2.97	Galloyl glucose	C_13_H_15_O_10_^−^	331.0671	331.0669	−0.4	168.8574	[19]
2	10.06	Unknown	C_16_H_19_O_9_^−^	355.1035	355.1037	0.7		
3	10.17	Unknown	C_16_H_17_O_8_^−^	337.0929	337.0926	−0.8		
4	11.05	Unknown	C_9_H_7_O_3_^−^	163.0401	163.0401	0.3		
5	11.39	Dihydroquercetin 3-*O*-rhamnoside	C_21_H_21_O_11_^−^	449.1089	449.1087	−0.5	284.8040, 256.8297, 151.8702	FooDB FDB007557
6	11.45	Quercetin 3-*O*-glucoside	C_21_H_19_O_12_^−^	463.0882	463.0884	0.4	300.7885, 270.7986, 254.8152	[20]
7	11.6	Hesperidin	C_28_H_33_O_15_^−^	609.1825	609.1835	1.7	300.8283, 285.8131	[21]
8	11.77	Quercetin 3-*O*-pentoside	C_20_H_17_O_11_^−^	433.0776	433.0774	−0.4	299.7795	[22]
9	12.04	Quercetin 3-*O*-rhamnoside	C_21_H_19_O_11_^−^	447.0933	447.0929	−0.9	300.7888, 270.7983, 254.8150, 150.8639	[23]
10	12.25	Isorhamnetin	C_16_H_11_O_7_^−^	315.0510	315.0510	−0.1	270.8001, 242.8017, 226.8453	FooDB FDB002422
11	12,67	Kaempferol 3-*O*-rhamnoside	C_21_H_19_O_10_^−^	431.0984	431.0985	0.4	284.8040	FooDB FDB01691
12	12.96	Delphinidin 3-*O*-(6-*p*-coumaroyl-glucoside)	C_30_H_27_O_14_^+^	611.1328	611.1300	−4.7	302.7946, 272.7952, 255.8279, 151.8627	[24]
13	13.09	Pelargonidin-3-*O*-beta-*D*-*p*-coumaroylglucoside	C_30_H_26_O_12_^+^	578.1352	578.1355	0.6	431.7574, 413.7599, 271.8191	FooDB FDB031089
14	13.36	Cyanidin 3-(*p*-coumaroyl)-glucoside	C_30_H_27_O_13_^+^	595.1301	595.1306	0.9	286.8026, 254.8140	Pubchem 102514898
15	15.28	Vanillic acid	C_8_H_7_O_4_^−^	167.0350	167.0350	0.4	136.8979, 108.9227	FooDB FDB0008416
16	15.58	Unknown	C_39_H_59_O_5_^−^	607.4368	607.4365	−0.5	264.9243, 96.8608	
17	16.17	Carvacrol	C_10_H_13_O^−^	149.0972	149.0971	−0.5	116.9226, 105.0340, 93.0704	FooDB FDB013868
18	16.53	Eupatorin	C_18_H_15_O_7_^−^	343.0823	343.0821	−0.7	214.8393	[25]

**Table 2:** Identification of metabolites in the aqueous fraction of *E. cordifolia* leaves.

Peak	Retention Time (min)	Tentative Identification	Molecular Formula	Theoretical Mass (*m*/*z*)	Measured Mass [M−H]^−^ (*m*/*z*)	Accuracy (ppm)	MS^n^ Ions	Reference
1	7.51	Procyanidin dimer B1	C_30_H_25_O_12_^−^	577.1352	577.1366	2.5	406.7550, 288.8324, 124.9046	[26]
2	7.64	Procyanidin trimer EEC	C_45_H_37_O_18_^−^	865.1985	865.2006	2.4	-	[27]
3	11.51	Dihydroquercetin 3-*O*-rhamnoside	C_21_H_21_O_11_^−^	449.1089	449.1085	−0.9	284.8044, 270.7905, 151.8745	FooDB FDB007557
4	11.57	Quercetin 3-*O*-glucoside (Isoquercetin)	C_21_H_19_O_12_^−^	463.0882	463.0874	−1.8	300.7866, 270.7982, 254.8142, 150.8548	[20]
5	11.72	Hesperidin	C_28_H_33_O_15_^−^	609.1825	609.1830	0.8	300.8245, 285.8117, 256.8657	[21]
6	11.86	Quercetin 3-*O*-pentoside	C_20_H_17_O_11_^−^	433.0776	433.0776	−0.1	-	[22]
7	12.00	Quercetin 3-*O*-rhamnoside	C_21_H_19_O_11_^−^	447.0933	447.0925	−1.8	300.7885, 270.7970, 254.8149, 150.8641	[23]
8	14.93	Vanillic acid	C_8_H_7_O_4_^−^	167.0350	167.0356	4.0	136.8934, 108.9212	PubChem 8468
9	15.10	Cyanidin 3-(*p*-coumaroyl)-glucoside	C_30_H_27_O_13_^+^	595.1301	595.1297	−0.7	286.8026, 254.8140	Pubchem 102514898
10	15.31	Unknown	C_9_H_11_O_2_^−^	151.0765	151.0764	−0.2	-	
11	15.65	Unknown	C_39_H_59_O_5_^−^	607.4368	607.4360	−1.2	264.9257, 96.8624	
12	16.25	Carvacrol	C_10_H_13_O^−^	149.0972	149.0971	−0.6	116.9226, 133.1017 105.0340	PubChem 10364

**Table 3:** Identification of metabolites in the EtOAc extract of flowers.

Peak	Retention Time (min)	Tentative Identification	Molecular Formula	Theoretical Mass (*m*/*z*)	Measured Mass [M−H]^−^ (*m*/*z*)	Accuracy (ppm)	MS^n^ Ions	Reference
1	2.14	3-Feruloylquinic acid	C_17_H_19_O_9_^−^	367.1035	367.1049	3.9	172.8436, 112.9139, 94.9186	FooDB FDB000249
2	2.93	Galloyl glucose	C_13_H_15_O_10_^−^	331.0671	331.0669	−0.5	168.8608	[19]
3	4.18	Unknown	C_6_H_5_O_3_^−^	125.0244	125.0244	−0.3	-	
4	7.25	Resorcinol	C_6_H_5_O_2_^−^	109.0295	109.0292	−2.4	-	PubChem 5054
5	8.35	(-)-Catechin	C_15_H_13_O_6_^−^	289.0718	289.0717	−0.1	149.8930, 122.9261	[27]
6	9.54	Procyanidin trimer EEC	C_45_H_37_O_18_^−^	865.1985	865.1995	1.1	-	[27]
7	10.08	Ferulic acid 4-*O*-glucoside	C_16_H_19_O_9_^−^	355.1035	355.1034	−0.1	160.8772, 132.9035	FooDB FDB000256
8	10.19	5-*p*-Coumaroylquinic acid	C_16_H_17_O_8_^−^	337.0929	337.0937	2.4	-	FooDB FDB000236
9	10.73	Unknown	C_22_H_17_O_10_^−^	441.0827	441.0822	−1.1	-	
10	10.89	Isorhamnetin 3-*O*-glucoside	C_22_H_21_O_12_^−^	477.1039	477.1039	0.1	313.7841, 271.8078	PubChem 5318645
11	11.18	Unknown	C_30_H_23_O_12_^−^	575.1195	575.1198	0.5	406.7520	
12	11.4	Bis-8,8-Catechinyl methane	C_31_H_27_O_12_^−^	591.1508	591.1508	0.0	288.8312, 244.8721, 136.8944	PubChem 46182787
13	11.41	Dihydroquercetin 3-*O*-rhamnoside	C_21_H_21_O_11_^−^	449.1089	449.1087	−0.5	284.8020, 216.8605, 150.8997	FooDB FDB007557
14	11.47	Quercetin 3-*O*-glucoside	C_21_H_19_O_12_^−^	463.0882	463.0881	−0.1	300.7875, 270.7981, 254.8146	[20]
15	11.79	Quercetin 3-*O*-pentoside	C_20_H_17_O_11_^−^	433.0776	433.0775	−0.4	299.7797, 278.7976, 254.8143	[22]
16	12.06	Quercetin 3-*O*-rhamnoside	C_21_H_19_O_11_^−^	447.0933	447.0929	−0.9	300.7869, 270.7972, 254.8137, 150.8626	[23]
17	12.27	Ishormanetin	C_16_H_11_O_7_^−^	315.0510	315.0507	−1.2	270.7969, 242.8214, 226.8384	FooDB FDB002422
18	12.28	Kaempferol 3-*O*-arabinoside	C_20_H_17_O_10_^−^	417.0827	417.0824	−0.7	283.7958, 254.8146, 226.8385	PubChem 5481882
19	12.7	Kaempferol 3-*O*-rhamnoside	C_21_H_19_O_10_^−^	431.0984	431.0986	0.4	284.8033, 254.8159, 226.8400	FooDB FDB01691
20	12.84	Unknown	C_23_H_21_O_12_^−^	489.1039	489.1050	2.3	-	
21	13.11	Pelargonidin-3-*O*-beta-D-*p*-coumaroylglucoside	C_30_H_26_O_12_^+^	578.1352	578.1353	0.3	431.7581, 413.7604, 271.8189	
22	13.12	Unknown	C_26_H_31_O_14_^−^	567.1719	567.1718	−0.3	458.7602, 149.8549	
23	13.36	Petunidin 3-*O*-(6-*p*-coumaroyl-glucoside)	C_31_H_29_O_14_^−^	625.1485	625.1455	−4.7	316.7941, 299.7759	FooDB FDB000041
24	15.22	Vanillic acid	C_8_H_7_O_4_^−^	167.0350	167.0350	0.4	136.8979, 108.9227	FooDB FDB0008416
25	15.59	Unknown	C_39_H_59_O_5_^−^	607.4368	607.4365	−0.5	264.9243	
26	16.18	Carvacrol	C_10_H_13_O^−^	149.0972	149.0971	−0.5	93.0704	FooDB FDB013868
27	16.51	Eupatorin	C_18_H_15_O_7_^−^	343.0823	343.0821	−0.7	214.8393	[25]

**Table 4:** Identification of metabolites in the MeOH extract of flowers.

Peak	Retention Time (min)	Tentative Identification	Molecular Formula	Theoretical Mass (*m*/*z*)	Measured Mass [M−H]^−^ (*m*/*z*)	Accuracy (ppm)	MS^n^ Ions	Reference
1	4.25	Gallic acid	C_7_H_5_O_5_^−^	169.0142	169.0143	0.3	125.0244, 94.9150	FooDB FDB000662
2	7.39	Resorcinol	C_6_H_5_O_2_^−^	109.0295	109.0296	0.6	107.9144, 90.9250	PubChem 5054
3	8.44	(-)-Catechin	C_15_H_13_O_6_^−^	289.0718	289.0717	−0.1	149.8930, 122.9261	[27]
4	10.18	Ferulic acid 4-*O*-glucoside	C_16_H_19_O_9_^−^	355.1035	355.1034	−0.1	160.8772, 132.9035	FooDB FDB000256
5	10.19	Bis-8,8-Catechinylmethane	C_31_H_27_O_12_^−^	591.1508	591.1508	0.0	288.8312, 244.8721, 136.8944	
6	10.28	5-*p*-Coumaroylquinic acid	C_16_H_17_O_8_^−^	337.0929	337.0927	−0.7	190.8914, 92.9427	FooDB FDB000236
7	11.18	*p*-Coumaric acid	C_9_H_7_O_3_^−^	163.0401	163.0401	0.5	118.9340, 92.9390	FooDB FDB002593
8	11.29	Unknown	C_30_H_23_O_12_^−^	575.1195	575.1200	0.9	-	
9	11.52	Dihydroquercetin 3-*O*-rhamnoside	C_21_H_21_O_11_^−^	449.1089	449.1087	−0.5	284.8020, 216.8605, 150.8997	FooDB FDB007557
10	11.58	Dihydroquercetin 3-*O*-glucoside	C_21_H_21_O_12_^−^	465.1039	465.1039	0.1	301.7841, 150.8616	FooDB FDB021739
11	11.59	Quercetin 3-*O*-glucoside	C_21_H_19_O_12_^−^	463.0882	463.0881	−0.1	300.7875, 270.7981, 254.8146	[20]
12	11.9	Quercetin 3-*O*-pentoside	C_20_H_17_O_11_^−^	433.0776	433.0775	−0.4	299.7797, 278.7976, 254.8143	[22]
13	12.18	Quercetin 3-*O*-rhamnoside	C_21_H_19_O_11_^−^	447.0933	447.0929	−0.9	300.7869, 270.7972, 254.8137, 150.8626	[23]
14	12.41	Isorhamnetin	C_16_H_11_O_7_^−^	315.0510	315.0509	−0.3		FooDB FDB000604
15	12.59	Isorhamnetin 3-*O*-glucoside	C_22_H_21_O_12_^−^	477.1039	477.1036	−0.5		[28]
16	12.8	Kaempferol 3-*O*-rhamnoside	C_21_H_19_O_10_^−^	431.0984	431.0986	0.4	284.8033, 254.8159, 226.8400	FooDB FDB01691
17	13.02	Unknown	C_23_H_21_O_7_^−^	409.1293	409.1290	−0.6	216.8965, 160.9086, 1498904	
18	13.08	Delphinidin 3-*O*-(6-*p*-coumaroyl-glucoside)	C_30_H_26_O_14_^−^	610.1328	610.1299	−4.7	301.7882, 150.8636	[29]
19	13.12	Homovanillyl alcohol	C_9_H_11_O_3_^−^	167.0714	167.0714	0.0	121.9223, 107.9141	FooDB FDB018391
20	13.21	Pelargonidin-3-*O*-beta-*D*-*p*-coumaroylglucoside	C_30_H_26_O_12_^+^	578.1352	578.1359	1.2	431.7609, 270.8202, 144.8942	
21	13.42	Naringenin	C_15_H_11_O_5_^−^	271.0612	271.0614	0.7	118.9360, 106.9058, 92.9381	[30]
22	13.47	Petunidin 3-*O*-(6-*p*-coumaroyl-glucoside)	C_31_H_29_O_14_^−^	624.1485	624.1455	−4.7	477.7326, 315.7944, 299.7743	FooDB FDB000041
23	13.97	Apigenin	C_15_H_9_O_5_^−^	269.0455	269.0457	0.7	148.8828, 120.9104, 116.9188, 106.9067	[31]
24	14.83	Pinocembrin	C_15_H_11_O_4_^−^	255.0663	255.0661	−0.7	184.8977, 144.9281	[32]
25	15.06	Unknown	C_20_H_17_O_6_^−^	353.1031	353.1034	0.9	248.8433, 220.8707, 96.8616	
26	16.62	Eupatorin	C_18_H_15_O_7_^−^	343.0823	343.0821	−0.7	214.8563	[25]

**Table 5:** Identification of metabolites in the honey extract.

Peak	Retention Time (min)	Tentative Identification	Molecular Formula	Theoretical Mass (*m*/*z*)	Measured Mass [M−H]^−^ (*m*/*z*)	Accuracy (ppm)	MS^n^ Ions	Reference
1	10.13	*p*-Anisaldehyde	C_8_H_7_O_2_^−^	135.0452	135.0452	0.6	88.9467	PubChem 31244
2	10.31	Syringaldehyde	C_9_H_9_O_4_^−^	181.0506	181.0508	1.0	120.9122, 91.9305	FooDB FDB000822
3	10.69	Myricetin 3-*O*-rutinoside	C_27_H_29_O_17_^−^	625.1410	625.1413	0.4	299.7784, 270.7989	FooDB FDB016619
4	11.29	Quercetin 3-*O*-rutinoside	C_27_H_29_O_16_^−^	609.1461	609.1455	−1.1	301.0351, 283.7972, 254.8131	[28]
5	11.73	Dihydroquercetin 3-*O*-rhamnoside	C_21_H_21_O_11_^−^	449.1089	449.1072	−3.9	284.8033, 150.8959	FooDB FDB007557
6	12.24	Mellein	C_10_H_9_O_3_^−^	177.0557	177.0558	0.5	116.9188, 104.9287	FooDB FDB000337
7	12.38	Quercetin 3-*O*-rhamnoside	C_21_H_19_O_11_^−^	447.0933	447.0920	−2.9	300.7859, 270.7964, 254.8124, 150.8624	[23]
8	13.36	Eugenol	C_10_H_11_O_2_^−^	163.0765	163.0767	1.8	147.0452	FooDB FDB012171
9	13.37	Homovanillyl alcohol	C_9_H_11_O_3_^−^	167.0714	167.0715	0.9	122.9267, 108.9180	FooDB FDB018391
10	13.43	Quercetin	C_15_H_9_O_7_^−^	301.0354	301.0351	−1.0	150.8646, 120.5446	[34]
11	13.6	Unknown	C_16_H_13_O_5_^−^	285.0768	285.0770	0.5	251.8283	
12	13.61	Formononetin	C_16_H_11_O_4_^−^	267.0663	267.0664	0.3	250.8207, 194.8696, 166.8975	FooDB FDB012219
13	13.78	Isorhamnetin	C_16_H_11_O_7_^−^	315.0510	315.0502	−2.6	270.7952, 254.8131, 242.8218	FooDB FDB002422
14	13.89	Naringenin	C_15_H_11_O_5_^−^	271.0612	271.0614	0.6	118.936, 106.9058, 92.9381	FooDB FDB000678
15	14.44	Kaempferide	C_16_H_11_O_6_^−^	299.0561	299.0559	−0.7	254.8137, 226.8389	FooDB FDB016499
16	14.96	Umbelliferone	C_9_H_5_O_3_^−^	161.0244	161.0245	0.7	143.0502, 133.0303	[35]
17	15.01	Cirsimaritin	C_17_H_13_O_6_^−^	313.0718	313.0717	−0.1	252.8350, 207.8788, 196.8851	[35]
18	15.06	3,7-Dimethyl quercetin	C_17_H_13_O_7_^−^	329.0667	329.0667	0.2	298.7730, 270.7949	[35]
19	15.07	Pinocembrin	C_15_H_11_O_4_^−^	255.0663	255.0665	0.9	212.8693, 184.8948	FooDB FDB002758
20	15.29	Daidzein	C_15_H_9_O_4_^−^	253.0506	253.0509	0.9	252.8379, 207.8706, 179.8956, 166.8974	FooDB FDB
21	15.37	Cyanidin 3-(*p*-coumaroyl)-glucoside	C_30_H_27_O_13_^+^	595.1301	595.1297	−3.3	286.8026, 254.8140	Pubchem 102514898
22	15.44	Apigenin	C_15_H_9_O_5_^−^	269.0455	269.0456	0.1	268.8264, 251.8329, 226.8345, 170.8903	[31]
23	15.93	Unknown	C_39_H_59_O_5_^−^	607.4368	607.4354	−2.4	264.8231, 96.8628	
24	16.8	Eupatorin	C_18_H_15_O_7_^−^	343.0823	343.0813	−3.1	214.8398	[25]

**Table 6:** Quantitation of compounds in *E. cordifolia* ethyl acetate, aqueous fraction, and methanolic extract of honey (mg/kg).

Compound	EtOAc F. Leaves	Aqueous F. Leaves	EtOAc E. Flower	MeOH E. Flower	UH055	UH056	UH057
Gallic acid	36.39 ± 0.41	173.02 ± 0.23	33.05 ± 0.22	1378.69 ± 0.38	15.78 ± 0.19	32.80 ± 0.20	3.72 ± 0.16
Cinnamic acid	nd	nd	nd	nd	d	d	d
Syringic acid	nd	nd	nd	nd	0.01	0.02	0.02
Ferulic acid	0.01	nd	0.01	nd	0.02	0.03	0.02
Chlorogenic acid	nd	0.25 ± 0.02	0.01 ± 0.01	nd	nd	nd	0.01 ± 0.01
Caffeic acid	0.03 ± 0.01	nd	0.03 ± 0.01	0.08 ± 0.01	0.04 ± 0.01	0.17 ± 0.01	0.12 ± 0.01
*p*-coumaric acid	0.02 ± 0.01	nd	0.07 ± 0.01	0.28 ± 0.01	0.05 ± 0.01	0.13 ± 0.01	0.06 ± 0.01
Benzoic acid	nd	nd	d	d	d	d	d
Pinocembrin	1.02 ± 0.08	nd	10.28 ± 0.09	123.24 ± 0.15	83.60 ± 0.16	1196.53 ± 0.24	231.44 ± 0.09
Rutin	nd	0.05 ± 0.01	0.02 ± 0.01	nd	0.02 ± 0.01	0.02 ± 0.01	0.01 ± 0.01
Quercetin	1.20 ± 0.02	nd	0.38 ± 0.01	3.57 ± 0.02	0.06 ± 0.01	0.06 ± 0.01	nd
Luteolin	0.73 ± 0.02	0.19 ± 0.02	0.13 ± 0.02	0.45 ± 0.02	0.02 ± 0.01	0.02 ± 0.01	0.02 ± 0.01
Kaempferol	0.05 ± 0.01	nd	0.04 ± 0.01	0.21 ± 0.01	0.05 ± 0.01	0.07 ± 0.01	0.03 ± 0.01
Epicatechin	19.72 ± 0.08	17.22 ± 0.06	0.19 ± 0.02	3.69 ± 0.02	0.25 ± 0.01	nd	0.04 ± 0.01
Catechin	13.06 ± 0.07	5.40 ± 0.04	0.12 ± 0.02	1.19 ± 0.01	0.10 ± 0.01	0.04 ± 0.01	0.03 ± 0.01
Apigenin	0.04 ± 0.01	nd	0.02 ± 0.01	0.07 ± 0.01	0.02 ± 0.01	0.03 ± 0.01	0.02 ± 0.01
Myricetin	0.03 ± 0.01	nd	0.02 ± 0.01	0.04 ± 0.01	nd	nd	nd
Isorhamnetin	42.55 ± 0.06	nd	2.44 ± 0.03	7.58 ± 0.04	1.39 ± 0.02	2.89 ± 0.02	1.16± 0.02
Taxifolin	d	d	d	d	d	d	d
Chrysin	d	nd	d	d	d	d	d
Galanganin	nd	nd	nd	d	d	d	d
Genistein	d	d	d	d	d	d	d
Hesperetin	d	d	d	d	d	d	d

d: detected; nd: not detected.

**Table 7:** Content of phenols and antioxidant activity of the different extracts of *E. cordifolia*.

Fractions/Extract	Total Phenolic Content (mg GAE/g DE)	DPPH		ABTS		FRAP (mmol Fe/g)
		(µmol TE/g DE)	CI_50_ (µg/mL)	(µmol TE/g DE)	CI_50_ (µg/mL)	
EtOAcF. leaves	302.19 ± 0.71 ^a^	174.91 ± 0.59 ^c,d^	45.05 ± 1.58 ^c^	146.74 ± 0.16 ^b^	41.25 ± 0.46 ^c^	2.74 ± 0.02 ^a^
Aqueous F. leaves	184.82 ± 11.02 ^b^	174.25 ± 1.23 ^d^	40.16 ± 1.66 ^d^	147.16 ± 0.08 ^b^	39.36 ± 1.65 ^d^	2.66 ± 0.04 ^b^
Ethyl acetate E. flowers	85.57 ± 2.21 ^c^	240.79 ± 6.75 ^b^	281.83 ± 5.28 ^a^	182.73 ± 3.23 ^a^	127.48 ± 5.36 ^a^	0.77 ± 0.04 ^d^
Methanol E. flowers	185.79 ± 10.19 ^b^	279.46 ± 8.17 ^a^	109.74 ± 1.74 ^b^	185.25 ± 4.31 ^a^	46.40 ± 3.60 ^b^	1.99 ± 0.30 ^c^
UH055	40.0 ± 2.0 ^e^	94.0 ± 3.0 ^f^	-	75 ± 7.0 ^d^	-	0.49 ± 0.02 ^f^
UH056	58.0 ± 5.0 ^d^	120.0 ± 3.0 ^e^	-	114 ± 15 ^c^	-	0.23 ± 0.01 ^g^
UH057	35.0 ± 2.0 ^e^	187.0 ± 3.0 ^c^	-	122 ± 13 ^c^	-	0.59 ± 0.02 ^e^

Different letters in the same column indicate significant differences: *p* < 0.05, *n* = 3. -: not evaluated.

## Data Availability

Raw HPLC MS data is available upon request.

## Supplementary Materials

The following supporting information can be downloaded at: https://www.mdpi.com/article/10.3390/antiox14030292/s1. Figure S1: Total ionic current chromatogram (TIC) in EtOAc fraction of *E. cordifolia* leaves. Figure S2: Total ionic current chromatogram (TIC) in the aqueous fraction of *E. cordifolia* leaves. Figure S3: Total ionic current chromatogram (TIC) in EtOAc extract of flowers of *E. cordifolia* leaves. Figure S4: Total ionic current chromatogram (TIC) in MeOH extract of flowers of *E. cordifolia* leaves. Figure S5: Total ionic current chromatogram (TIC) in honey extract: UH055 (a), UH056 (b) and UH057 (c).
