# Supplementary material for: Metabolomic Profiling and Antioxidant Properties of Chilean Eucryphia cordifolia Cav.: Insights from Leaves, Flowers, and Monofloral Honey
Source: Antioxidants (Basel). 2025 Feb 28;14(3):292. doi: 10.3390/antiox14030292 (PMC11939269; doi:10.3390/antiox14030292)
Supplement: Supplementary file 1 [file antioxidants-14-00292-s001.zip › antioxidants-3443181-Supplementary Materials.pdf]

Supplementary Materials

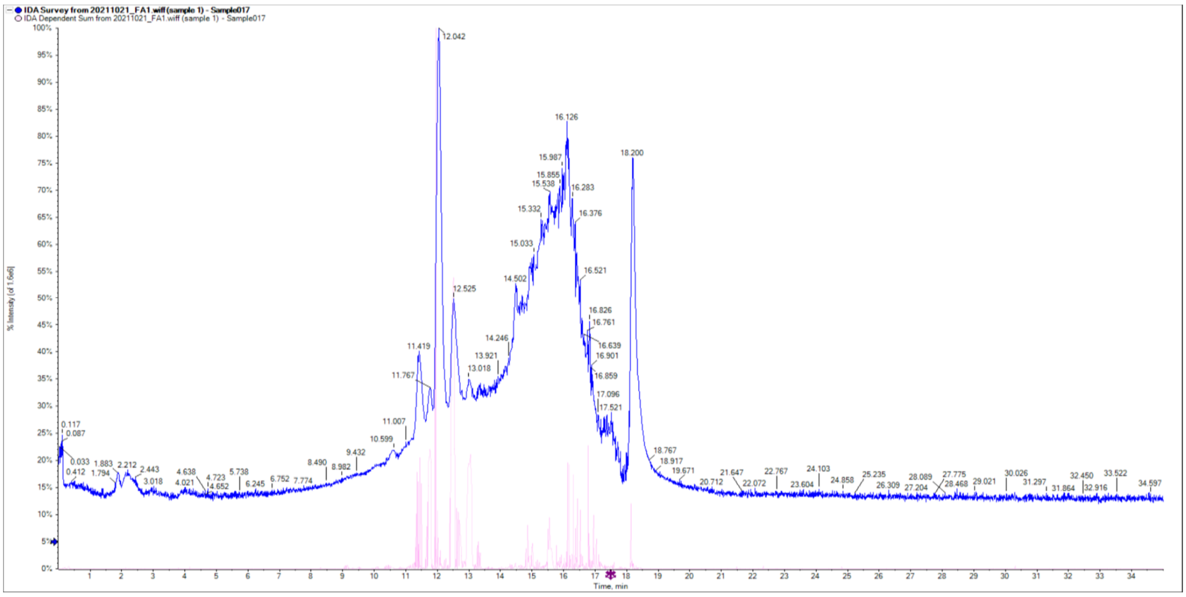

Figure S1. Typical chromatograms of negative mode extract analyzed by UHPLC/Q-TOF-MS: Total ionic current chromatogram (TIC) in EtOAc fraction of E. cordifolia leaves.

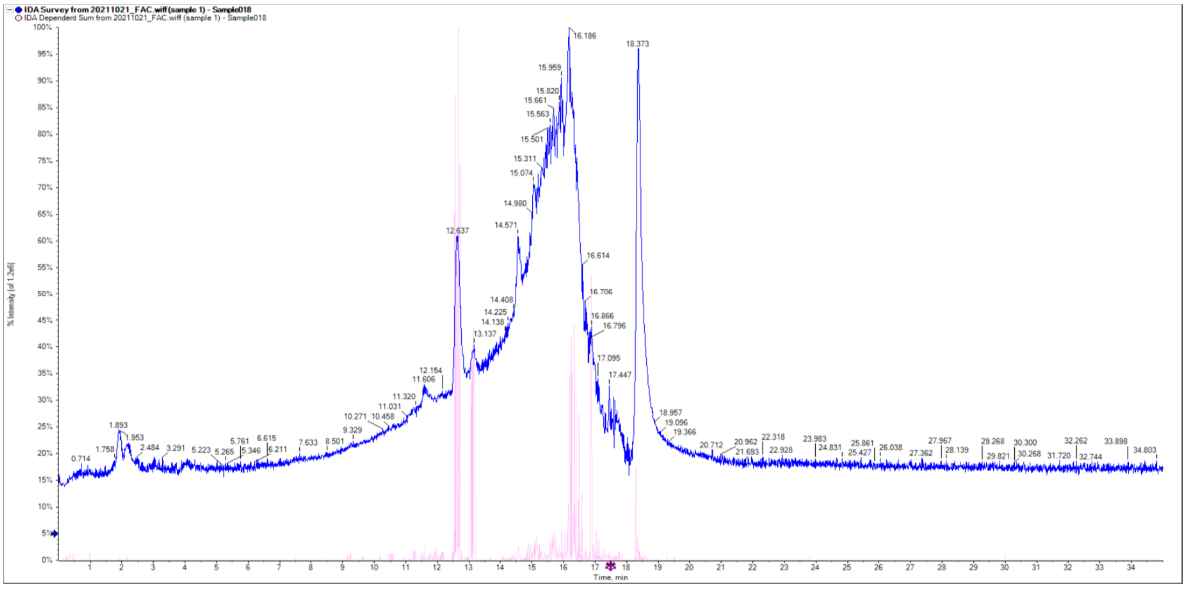

Figure S2. Typical chromatograms of negative mode extract analyzed by UHPLC/Q-TOF-MS: Total ionic current chromatogram (TIC) in the aqueous fraction of E. cordifolia leaves.

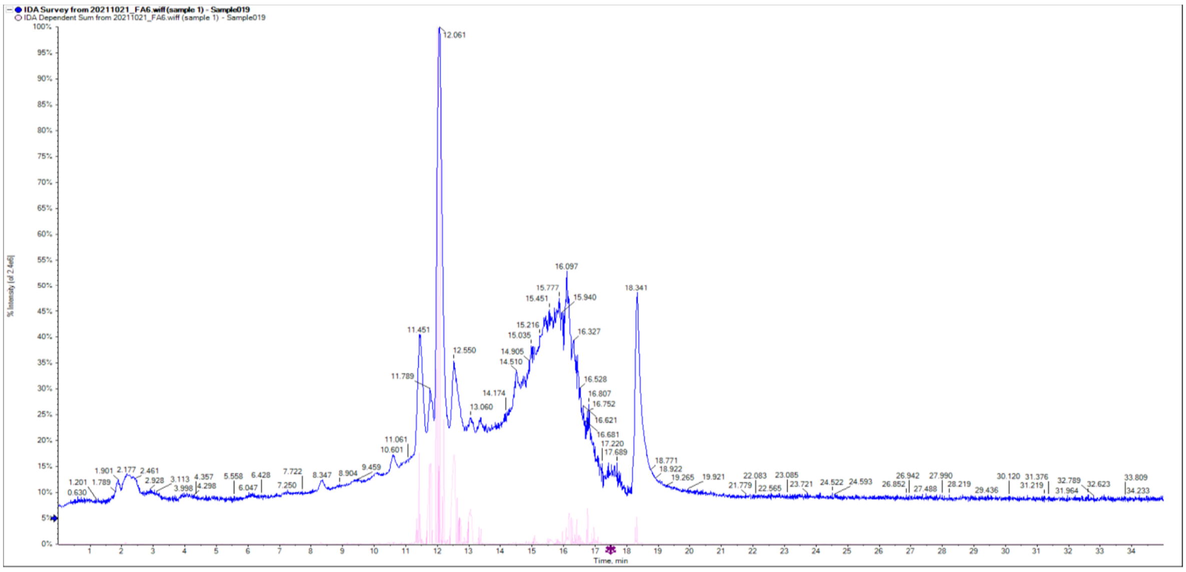

Figure S3. Typical chromatograms of negative mode extract analyzed by UHPLC/Q-TOF-MS: Total ionic current chromatogram (TIC) in EtOAc extract of flowers of E. cordifolia leaves.

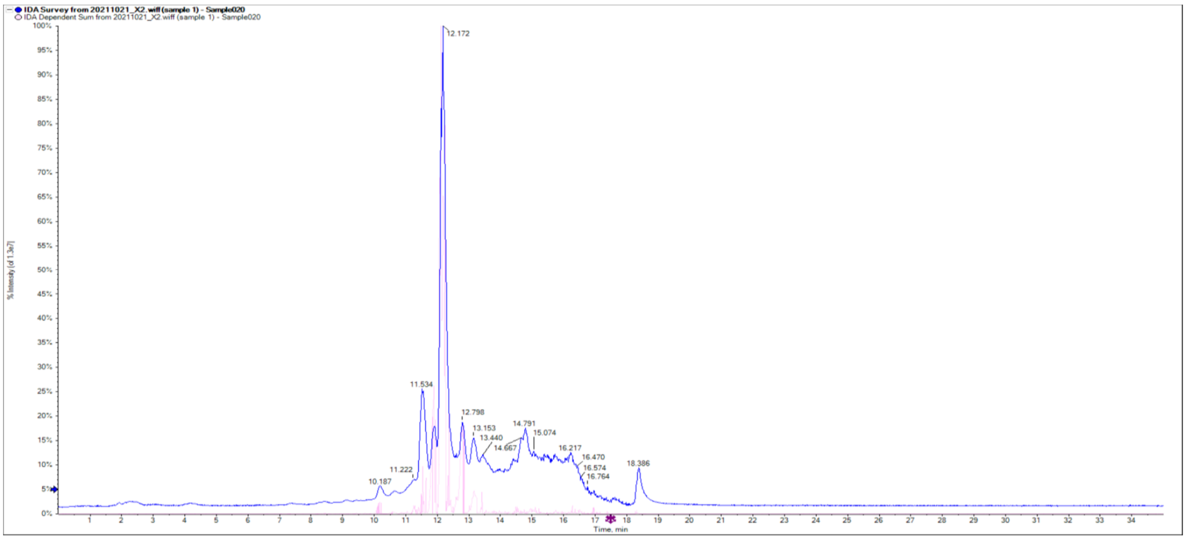

Figure S4. Typical chromatograms of negative mode extract analyzed by UHPLC/Q-TOF-MS: Total ionic current chromatogram (TIC) in MeOH extract of flowers of *E. cordifolia* leaves.

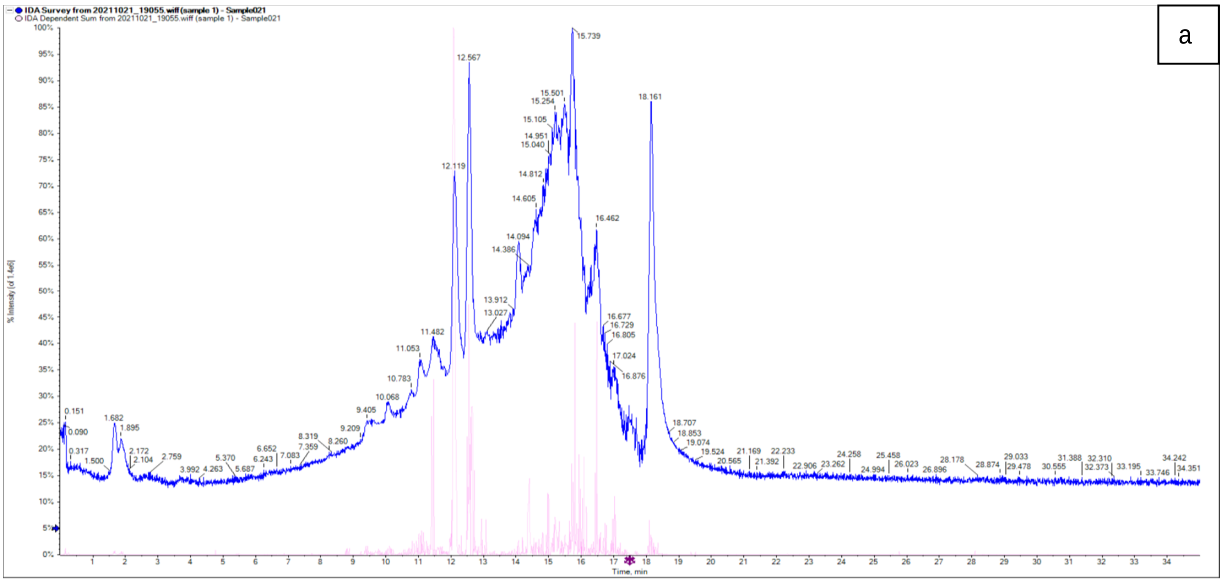
IDA Survey from 20211021_19055.wiff (sample 1) - Sample021
IDA Dependent Sum from 20211021_19055.wiff (sample 1) - Sample021
a
Time, min

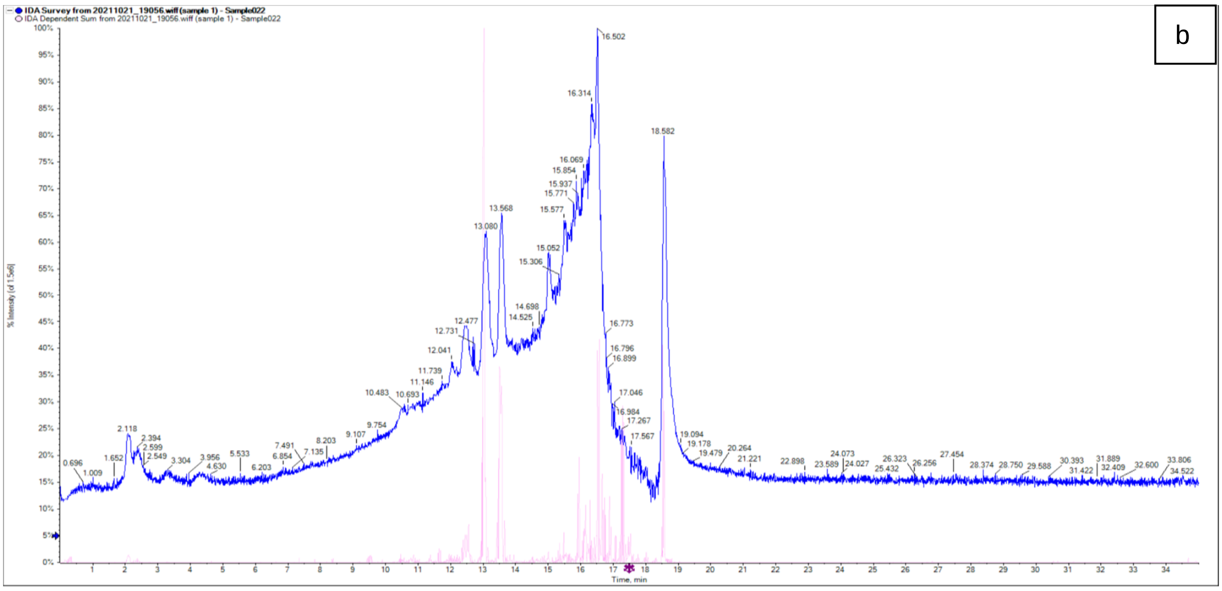
IDA Survey from 20211021_19056.wiff (sample 1) - Sample022
IDA Dependent Sum from 20211021_19056.wiff (sample 1) - Sample022
b
Time, min

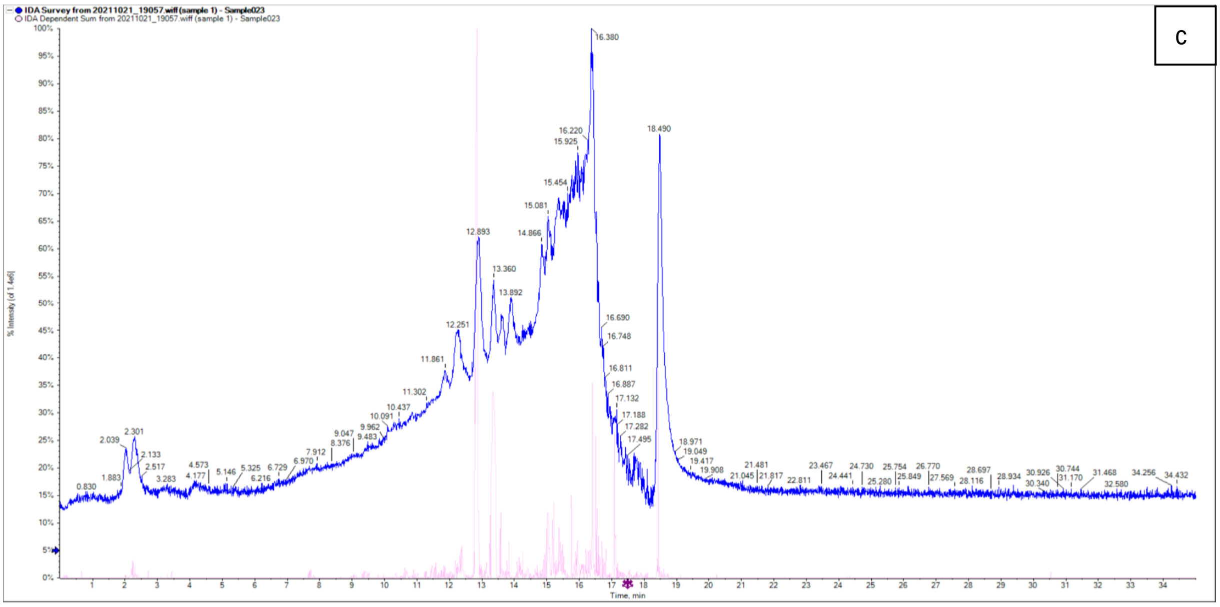
IDA Survey from 20211021_19057.wiff (sample 1) - Sample023
IDA Dependent Sum from 20211021_19057.wiff (sample 1) - Sample023
c
Time, min

Figure S5. Typical chromatograms of negative mode extract analyzed by UHPLC/Q-TOF-MS: Total ionic current chromatogram (TIC) in honey extract: UH055 (a), UH056 (b) and UH057 (c).
